# Resting state cortico-cerebellar functional connectivity networks: a comparison of anatomical and self-organizing map approaches

**DOI:** 10.3389/fnana.2012.00031

**Published:** 2012-08-10

**Authors:** Jessica A. Bernard, Rachael D. Seidler, Kelsey M. Hassevoort, Bryan L. Benson, Robert C. Welsh, Jillian Lee Wiggins, Susanne M. Jaeggi, Martin Buschkuehl, Christopher S. Monk, John Jonides, Scott J. Peltier

**Affiliations:** ^1^Department of Psychology, University of Michigan, Ann ArborMI, USA; ^2^School of Kinesiology, University of Michigan, Ann ArborMI, USA; ^3^Neuroscience Program, University of Michigan, Ann ArborMI, USA; ^4^Department of Radiology, University of Michigan, Ann ArborMI, USA; ^5^Department of Psychiatry, University of Michigan, Ann ArborMI, USA; ^6^Department of Psychology, University of Maryland, College ParkMD, USA; ^7^Functional MRI Laboratory, University of Michigan, Ann ArborMI, USA; ^8^Biomedical Engineering, University of Michigan, Ann ArborMI, USA

**Keywords:** cerebellum, resting state functional connectivity, self-organizing map

## Abstract

The cerebellum plays a role in a wide variety of complex behaviors. In order to better understand the role of the cerebellum in human behavior, it is important to know how this structure interacts with cortical and other subcortical regions of the brain. To date, several studies have investigated the cerebellum using resting-state functional connectivity magnetic resonance imaging (fcMRI; Krienen and Buckner, 2009; O'Reilly et al., 2010; Buckner et al., 2011). However, none of this work has taken an anatomically-driven lobular approach. Furthermore, though detailed maps of cerebral cortex and cerebellum networks have been proposed using different network solutions based on the cerebral cortex (Buckner et al., 2011), it remains unknown whether or not an anatomical lobular breakdown best encompasses the networks of the cerebellum. Here, we used fcMRI to create an anatomically-driven connectivity atlas of the cerebellar lobules. Timecourses were extracted from the lobules of the right hemisphere and vermis. We found distinct networks for the individual lobules with a clear division into “motor” and “non-motor” regions. We also used a self-organizing map (SOM) algorithm to parcellate the cerebellum. This allowed us to investigate redundancy and independence of the anatomically identified cerebellar networks. We found that while anatomical boundaries in the anterior cerebellum provide functional subdivisions of a larger motor grouping defined using our SOM algorithm, in the posterior cerebellum, the lobules were made up of sub-regions associated with distinct functional networks. Together, our results indicate that the lobular boundaries of the human cerebellum are not necessarily indicative of functional boundaries, though anatomical divisions can be useful. Additionally, driving the analyses from the cerebellum is key to determining the complete picture of functional connectivity within the structure.

## Introduction

The human cerebellum, while originally thought of as a sensorimotor structure, is now known to be as diverse in its functions as the cerebral cortex. For example, recent neuroimaging work has shown cerebellar activation associated with working memory and executive functions (Desmond et al., 2005; Kirschen et al., 2005; Hautzel et al., 2009; Stoodley and Schmahmann, 2009; Stoodley et al., 2012). These findings, coupled with evidence that the cerebellum plays a role in motor functions such as visuomotor learning (Imamizu et al., 2000; Inoue et al., 2000; Vaillancourt et al., 2006; Della-Maggiore et al., 2009), timing (Penhune et al., 1998; Spencer et al., 2003; Grube et al., 2010), and balance (Mauritz et al., 1979; Morton and Bastian, 2003; Sullivan et al., 2006), support the diverse functionality of the cerebellum. However, despite our advancing knowledge of cerebellar functions, the specific role this structure plays in these diverse behaviors remains unclear. Furthermore, how the human cerebellum interacts with the rest of the cortex also remains unclear, an understanding which may be crucial for shedding light on its specific functions.

Tract tracing investigations in non-human primates have provided insight into cerebellar-cortical interactions. The cerebellum has a distinct topography of cortical afferents and efferents. The anterior cerebellum (lobules IV, V, and VI), and lobule VIIIa form a network with the primary motor cortex, whereas posterior lobule Crus II has connections with area 46 of the monkey prefrontal cortex (Kelly and Strick, 2003). Interestingly, a similar dissociated topography of projections to motor and prefrontal cortex also exists in the dentate nucleus (Dum and Strick, 2003). Further, there are projections from the cerebellum to the basal ganglia (Hoshi et al., 2005) and vice versa (Bostan et al., 2010; for a review, see Bostan and Strick, 2010), and from the cerebellum to the posterior parietal cortex (Clower et al., 2005). Finally, the cerebellar vermis, once thought to receive inputs solely from the spinal cord, is now known to receive inputs from the motor cortex as well (Coffman et al., 2011). Thus, there are communication circuits between the cerebellum and a wide range of cortical and subcortical areas in the non-human primate, supporting its role in diverse motor and cognitive functions. In addition, this literature indicates a motor and cognitive topography within the cerebellum (for a review see Strick et al., 2009). It is notable that these data are based on animal models, and there are anatomical differences between the non-human primate cerebellum and the human cerebellum. However, they provide a guide for investigations of similar networks in the human brain.

Human neuroimaging has provided corroborating evidence of a functional topography in the cerebellum. Using meta-analysis, Stoodley and Schmahmann (2009) demonstrated that the cerebellar areas subserving motor functions are distinct from those subserving non-motor and cognitive functions. This has also been supported by functional MRI data indicating that motor tasks are largely the domain of the anterior cerebellum, whereas cognitive tasks, such as working memory, are supported by the posterior cerebellum (Stoodley et al., 2012).

Resting-state functional connectivity MRI (fcMRI) is an ideal method for investigating functional brain networks. It allows us to begin answering questions about interactions between the cerebellum and cerebral cortex in the human brain. Even when participants are not overtly performing an instructed task, there are low-frequency fluctuations in the blood-oxygen level dependent (BOLD) signal that exhibit correlations across multiple brain regions. Regions that perform similar functions show highly correlated activity while individuals are at rest (Biswal et al., 1995, 2010; for a review, see Fox and Raichle, 2007) revealing functional networks. These fluctuations in the resting-state BOLD signal are thought to be due to underlying fluctuations in neuronal activity (Shmuel and Leopold, 2008; Schölvinck et al., 2010). Using this technique, we are able to look at multiple networks at once, which allows for comparisons across these networks.

Several studies to date have harnessed fcMRI to investigate the human cerebellum, providing insight into cerebellar-cortical interactions (Allen et al., 2005; Habas et al., 2009; Krienen and Buckner, 2009; O'Reilly et al., 2010). Allen and colleagues (2005) demonstrated that the resting signal of the dentate nucleus as a whole is correlated with motor, prefrontal, and parietal cortices. Studies using fcMRI to study the cerebellar lobules have provided evidence to support the functional topography of the cerebellum seen in task-based literature (Krienen and Buckner, 2009; O'Reilly et al., 2010). Anterior portions of the cerebellum along with lobules VIIIa and VIIIb are part of motor networks, whereas the majority of the posterior regions of the cerebellum are part of networks including prefrontal and parietal cortices, associated most typically with cognitive functions. Finally, distinct cerebellar contributions to known cortical networks such as the default mode, salience, and executive networks have been delineated (Habas et al., 2009). Yet, despite this growing knowledge of cerebellar function and connectivity, we are still lacking a lobular based atlas of human cerebellar connectivity. Such an atlas provides an important point of comparison with the non-human primate literature, as well as the known human task-based functional MRI literature that indicates a functional topography. Furthermore, such an atlas is important in cases of disease or infarct, where specific lobules may be impacted. This would provide an important point of reference for understanding the functional networks potentially impacted in such an instance.

Recently, Buckner and colleagues (2011) conducted a large-scale investigation of resting state functional connectivity of the cerebellum based on previously delineated functional networks in the cerebral cortex (Yeo et al., 2011). They employed a winner-take-all approach to mapping the connectivity of each cerebellar voxel with known cortical networks, in addition to a demonstration of somatotopy in the motor networks of the anterior cerebellum. Their findings indicate that there are two, and possibly three, complete mappings of the cerebral cortex in a mirrored orientation within the cerebellum (Buckner et al., 2011). While this study provides us with great insight into the contributions of the cerebellum to known cortical networks, key questions remain. First, as Buckner and colleagues (2011) note, the winner-take-all approach leaves open the possibility that within a given region (or voxel) of the cerebellum, there may be correlations with multiple networks in the cerebral cortex that are not accounted for. By investigating cerebellar lobules, we can better understand their range of functional involvement. Second, Buckner and colleagues (2011) used cortical networks (either 7 or 17) to investigate cerebellar organization. However, it is possible that cerebello-thalamo-cortical networks involve a few, or even a single cortical structure. Taking an anatomical approach starting with regions of interest in the cerebellum allows us to identify such potential networks. Imposing a cortically-driven solution on the cerebellum, while logical and informative, ignores the existing clear anatomical organization that has been linked to precise functions in the nonhuman animal literature (Strick et al., 2009).

Here, we use fcMRI to investigate the networks of the cerebellar lobules and vermis. The purpose of this study is to compare the resting state networks of the cerebellum, as defined by lobular anatomy to those defined functionally, using a self-organizing map (SOM) algorithm. This allows us to investigate whether functional boundaries map onto anatomical boundaries in the human cerebellum. Taking a lobular anatomical approach allows us to investigate whether the anatomical breakdown of the cerebellum is a marker for distinct functional processing regions within the structure. Further, we can identify the range of functional involvement for each individual lobule as they may contribute to multiple resting-state networks. We hypothesize an anterior and posterior distinction between motor and cognitive regions of the cerebellum, with additional motor connectivity for lobule VIII. However, we predict that within the anterior and posterior regions, individual lobules will be part of distinct networks related to specific regions of the cortex, in line with the non-human primate literature. Furthermore, we also use a SOM algorithm to parcellate cerebellar networks. Several studies have investigated functional whole brain parcellations using a SOM approach [for example, see Beckmann et al. (2005) and Craddock et al. (2012)]. The algorithm parcellates the resting-state data based on similarities in the signal in all of the voxels of the brain structure in question. The resultant parcellations are used as masks to examine cerebello-cortical resting state functional connectivity. The networks produced through this self-organizing approach are compared to those from our anatomical analyses so as to determine whether functional divisions map onto lobular divisions of the cerebellum. There are several possible outcomes of this analysis. First, the SOM approach may produce clusters largely in line with individual cerebellar lobules, though larger lobules may be split into smaller sub-components, perhaps resulting in more focal functional networks than those seen with anatomical mapping alone. Second, the SOM approach is blind to anatomical borders, and may potentially cluster individual anatomical lobules together. Relatedly, the SOM approach may also reveal bilateral networks, wherein homologous lobules are grouped across the two cerebellar hemispheres.

## Methods

### Participants

We recruited 39 right-handed participants (Age ± stdev; 22.76 ± 2.95 years, 17 females) from the University of Michigan and greater Ann Arbor community. All participants were healthy, with no history of neurological or psychiatric disorder, and had no contraindications for fMRI scanning. Participants signed a consent form approved by the University of Michigan Medical Institutional Review Board. All participants were compensated $15 per hour for their participation. Three participants were excluded from analyses due to motion artifacts, and two participants were excluded due to technical problems during data collection, leaving a total of 33 (15 female) participants.

### fMRI data acquisition

Functional MRI data were collected with a 3T GE Signa scanner at the University of Michigan. A single-shot gradient-echo rev-erse spiral pulse sequence (Glover and Law, 2001) was used to collect either 300 (*n* = 12 participants) or 240 (*n* = 18 participants) T2^*^-weighted BOLD images (TR = 2 s, TE = 30 ms, flip angle = 90°, FOV = 220 × 220 mm, voxel size = 3.4 × 3.4 × 3.2 mm, and 40 axial slices). For the structural images, a 3D T1 axial overlay (TR = 8.9 ms, TE = 1.8 ms, flip angle = 15°, FOV = 260 mm, slice thickness = 1.4 mm, and 124 slices; matrix = 256 × 160) was acquired for anatomical localization. To facilitate normalization, a 110 slice (sagittal) inversion-prepped T1-weighted anatomical image using spoiled gradient-recalled acquisition in steady state (SPGR) imaging (flip angle = 15°, FOV = 260, 1.4 mm slice thickness) was acquired. A visual fixation cross was presented to the subject using a rear projection visual display. Participants were instructed to keep their eyes focused on the cross, and to not think about anything in particular. Data from multiple studies were combined, thus the duration of the scan was either 8 or 10 min. A pressure belt was placed on the abdomen of each subject to monitor the respiratory signal. A pulse oximeter was placed on the subject's finger to monitor the cardiac signal. The respiratory, cardiac, and fMRI data collection were synchronized.

### fMRI data analysis

The functional MRI data were preprocessed as part of the standard processing stream at the University of Michigan. K-space outliers in the raw data greater than two standard deviations from their mean were first replaced with the average of their temporal neighbors. Next, images were reconstructed using field map correction to remove distortions from magnetic field inhomogeneity. Third, physiological variations in the data from the cardiac and respiratory rhythms were removed via regression (Glover et al., 2000). This removed the effects of the first and second order harmonics of the externally collected physiological waveforms. Fourth, slice timing differences were corrected using local sinc interpolation (Oppenheim et al., 1999). Lastly, we used MCFLIRT in the fMRIB Software Library (Jenkinson et al., 2002) to perform motion correction (using the 10th image volume as the reference). For all participants, head motion was less than 0.5 mm in the x, y, or z direction (average = 0.09, 0.03, and 0.02 mm in the x, y, and z directions, respectively). Structural images were skull-stripped using FSL and we then registered the 3D T1 SPGR to the functional images using Advanced Normalization Tools (ANTS; Avants et al., 2008; Penn Image Computing and Science Lab, http://www.picsl.upenn.edu/ANTS/). The data were then normalized to MNI space using ANTS. The transformation was first applied to the SPGR image, and then the resulting warp vectors were applied to the functional images. Additionally, because of the potential for distortions when normalizing the cerebellum to standard space (Diedrichsen et al., 2009), the cerebellum was normalized separately to a spatially unbiased atlas template (SUIT; Diedrichsen, 2006; Diedrichsen et al., 2009) also using ANTS. Similarly, the warp vectors were then applied to the functional images resulting in normalized whole-brain structural and functional images, and separate normalized cerebellar structural and functional images. All data were smoothed using a 4 mm FWHM Gaussian smoothing kernel. Importantly, smoothing of the functional data in the cerebellum occurred *after* this data was normalized and removed from the whole brain.

### Functional connectivity analysis

Functional connectivity analysis was completed on all 33 participants. Because of the variable duration of the resting state scans, only the first 8 min of functional data were used in our analyses. The following procedures were used to generate functional connectivity maps (low frequency time course correlation maps). The data were first filtered using a second order dual-pass band-pass filter (cutoff frequencies = 0.001 and 0.16 HZ) to examine the band of interest (0–0.08 Hz) and to exclude higher frequency sources of noise such as heart rate and respiration (Biswal et al., 1995; Peltier et al., 2003).

Second, the time course of BOLD activity was extracted from each of the 10 lobules within the right cerebellar hemisphere and 8 lobules within the vermis, using masks created with the SUIT atlas (Diedrichsen et al., 2009) (see Figure 3A), creating an average timecourse for all of the voxels within the mask. For individuals where lobules were not included due to coverage of the cerebellum, these individuals were not included in the analysis for that particular lobule. The resultant sample size is indicated with the results. Notably, Lobules I, II, III, and IV are combined in the SUIT atlas so they were investigated together. They are referred to as Lobules I–IV. We were also unable to successfully create a mask for Vermis Crus I, and were therefore unable to include this lobule in our analyses. Third, the timecourse of the lobule was unit normalized to remove differences in mean and variance between spatial regions. Notably, these first three steps were completed on *unsmoothed* functional data to minimize contamination of the signal from neighboring lobules. Fourth, the average timecourse for each lobule in the filtered data was correlated with all other low-pass filtered voxels in both the cerebellum and the whole brain functional data (done in two separate steps using *smoothed* data) to form functional connectivity maps for each region of interest in each participant. Using Fisher's r-to-z transform, the correlations in the functional connectivity maps were converted to z-scores. The Z scores from each participant were then entered into the group-level analyses, which were carried out using SPM5 (Wellcome Department of Cognitive Neurology, London, UK; http://www.fil.ion.ucl.ac.uk). In our group analyses we used *t*-tests to assess whether the z-scores at the group level were significant. We evaluated the connectivity maps associated with the lobular regions of interest using a family-wise error correction of *p* < 0.001 (unless otherwise indicated) with a voxel extent threshold of at least 100 voxels (Nichols and Hayasaka, 2003). To confirm our ability to successfully map these networks, we also placed a single-voxel seed in the left primary motor cortex (*x*, *y*, and *z* coordinates −38, −16, and 40, respectively, in standardized space) and investigated connectivity with the cerebellum. We evaluated the results of this analysis using a family-wise error correction of *p* < 0.05 with a voxel extent threshold of at least 10 voxels.

Finally, to investigate the overall functional organization of the cerebellum using anatomical regions of interest, we created a correlation matrix. Here, we used the average timecourse in each lobule of the right hemisphere and the vermis to complete the correlations. Only data from participants with complete coverage of the cerebellum were included in this analysis (*n* = 19). This allowed us to investigate the extent to which anatomically derived cerebellar connectivity is based on local versus longer-range correlations.

### Self-organizing map algorithm

Only data from participants with complete coverage of the cerebellum were included in our analysis using the SOM algorithm. A subset of 19 participants was included in this analysis. Temporally filtered functional data from within the cerebellum only were entered into the algorithm.

An analysis of the functional organization of the cerebellum was implemented using the SOM algorithm, which has previously been used to analyze resting-state fMRI data (Peltier et al., 2003; Wiggins et al., 2011). The algorithm produces a topologically ordered feature map that represents the underlying probability density function of the data with minimal error (Kohonen, 1995). The exemplar matrix (initialized to random noise) is updated in successive iterations. The algorithm compares the data timecourses to every exemplar timecourse, using a prescribed distance metric to find the closest timecourse. The exemplars are then updated at each iteration using:

$$m_{i}\left(t+1\right)=m_{i}\left(t\right)+h_{\text{ci}}\left(t\right)*\left[x-m_{i}\left(t\right)\right]$$

where *t* is the current iteration number, and *h*_ci_(*t*) is a (time-dependent) neighborhood function that controls how many neighboring exemplars in addition to the closest exemplar are also updated, and to what degree. As the iterations progress, the neighborhood function shrinks the neighborhood; so while initially, the exemplar map receives global ordering, at the end, only individual nodes are updated.

In this study, a SOM algorithm was implemented with 100 exemplars (Peltier et al., 2003), using a correlation distance metric, with the neighborhood contraction rate implemented as a shrinking Gaussian neighborhood function, dependent on the iteration number (*t*):

$$h_{\text{ci}}\left(t\right)=\alpha \left(t\right)*\text{exp}\left(-{\left|\left|r_{i}-r_{c}\right|\right|}^{2}/\left(2*\sigma {\left(t\right)}^{2}\right)\right)$$

where α (*t*) is a learning rate that controls how fast the exemplars change, set to be 0.1 initially, decreasing by 0.5% at each iteration; *r*_i_ and *r*_c_ are the coordinates (in the two-dimensional exemplar matrix) of exemplars *i* and *c*; and σ (*t*) is the FWHM of the Gaussian function, initially set at five nodes to give global topographical ordering, but then decreasing by 4% at each iteration to switch to local ordering.

Timecourse data from all 19 subjects were unit-normalized and concatenated together, and the SOM algorithm was used to generate a set of exemplars. In order to reduce the amount of resultant data, the 100 exemplars in the final exemplar matrix were then grouped into superclusters, using a least-squares distance metric with no global ordering, to examine the principal groupings of the exemplars. The supercluster maps produced through the SOM algorithm were then used as seed regions for resting state functional connectivity analysis as described above. Results of our lobular based analysis and our analysis using the SOM algorithm to subdivide the cerebellum are described and compared below.

Finally, we ran two additional analyses to investigate the reproducibility and consistency of the number of clusters produced. First, we ran 1000 iterations of superclustering on the complete data set to investigate the consistency of the results. Second, the full data set was randomly divided into two separate groups (*n* = 10 and *n* = 9), and the SOM algorithm was run on these subsets, and 100 instances of superclustering was done on the resulting exemplar matrix for each group. This allowed us to investigate the reproducibility of the results.

## Results

### Lobular connectivity

We present connectivity results within the cerebellum separately from within the cortex because of our normalization procedures. Areas of correlation within the cerebellum were identified using the spatially unbiased atlas of the cerebellum (Diedrichsen, 2006; Diedrichsen et al., 2009). General patterns of results are described below. For complete lists of correlated cerebellar and cortical regions, as well as the number of participants used for the analyses of each lobule, please see Tables 1–4. Tables 1, 2 present the correlations for each lobule of the right hemisphere and vermis within the cerebellum, while Tables 3, 4 present the correlations between each lobule of the right hemisphere and vermis with the whole brain. In all the lobules, strong correlations were seen within the lobule itself. This is expected as we used an average timecourse across the lobules, and ran our analyses with every voxel within the brain.

**Table 1:** **MNI coordinates of the local maxima of cerebellar regions showing functional connectivity with the lobules of the right cerebellar hemisphere**.

**Seed**	**Region**	**MNI coordinates**			***T*-value**
		***x***	***y***	***z***	
Lobules I–IV^***^, (*n* = 33)	Lobules I–IV	10	−41	−19	19.28
	Crus I	40	−68	−34	11.17
Lobule V^***^, (*n* = 33)	Lobule V	14	−53	−11	23.43
	Lobule VIIIb	−18	−41	−48	11.18
	Crus II	10	−88	−33	10.77
	Lobule VIIIa	25	−56	−50	8.89
Lobule VI^***^, (*n* = 33)	Lobule VI	36	−48	−23	19.80
	Lobule VIIIa	27	−63	−53	11.00
	Lobule X	−18	−41	−47	10.68
	Crus I	−35	−65	−41	9.56
Crus I^***^, (*n* = 30)	Crus I	21	−86	−24	21.93
	Lobule IX	11	−53	−41	14.93
	Brainstem	10	−27	−33	12.66
	Lobule X	−18	−41	−47	10.13
	Lobules I–IV	−3	−50	−12	9.80
Crus II^***^, (*n* = 25)	Crus II	22	−88	−38	18.37
		−12	−90	−34	16.39
	Lobule IX	−6	−56	−52	12.52
		5	−53	−38	12.48
Lobule VIIb^**^, (*n* = 20)	Lobule VIIb	32	−72	−51	19.07
	Lobule VI	−6	−82	−19	14.93
		−25	−54	−26	14.12
		30	−59	−29	12.44
	Lobule IX	13	−57	−49	12.17
	Crus I	−38	−74	−22	11.38
Lobule VIIIa^**^, (*n* = 19)	Lobule VIIIa	32	−56	−55	18.81
		−28	−52	−52	13.17
	Lobule VI	−33	−59	−21	17.53
		33	−53	−20	13.58
		−8	−73	−14	11.33
	Lobule VIIb	26	−76	−51	12.74
	Lobule VIIIb	−20	−40	−50	12.59
Lobule VIIIb^†^, (*n* = 19)	Lobule VIIIb	18	−40	−54	17.13
		−15	−41	−54	11.25
	Lobule VIIIa	−29	−57	−61	11.24
		35	−59	−58	10.86
Lobule IX^*^, (*n* = 23)	Lobule IX	6	−56	−51	21.31
	Crus I	−31	−83	−27	16.82
	Lobule VI	−27	−48	−20	9.41
	Lobules I–IV	2	−49	−18	9.02
	Lobule VIIIa	−25	−53	−59	8.60
Lobule X^***^, (*n* = 33)	Lobule X	19	−38	−46	20.71
		−24	−34	−45	13.66
	Crus II	−4	−85	−28	11.10
		−23	−76	−39	9.39
	Lobule VI	−16	−62	−20	9.30
	Crus I	13	−83	−24	9.21

Negative x-values indicate locations in the left hemisphere, while positive x-values indicate locations in the right hemisphere. All results are family-wise error corrected,

† p < 0.05,

* p < 0.01,

** p < 0.005,

*** p < 0.001.

**Table 2:** **MNI coordinates of the local maxima of cerebellar regions showing functional connectivity with the lobules of the cerebellar vermis**.

**Seed**	**Region**	**MNI coordinates**			***T*-value**
		***x***	***y***	***z***	
Vermis VI^***^, (*n* = 33)	Vermis VI	2	−70	−17	17.98
	Brainstem	−3	−32	−43	10.35
		6	−24	4	9.06
	Crus II	31	−68	−43	9.64
	Vermis IX	3	−58	−37	9.29
Vermis Crus II^***^, (*n* = 33)	Vermis Crus II	0	−74	−32	32.07
	Lobule VI	−25	−65	−33	10.83
Vermis VIIb^**^, (*n* = 33)	Vermis VIIb	0	−68	−31	40.26
	Lobules I–IV	−1	−45	−21	10.39
		17	−38	−20	9.62
	Lobule V	3	−62	−9	9.26
	Lobule VI	20	−63	−14	9.10
Vermis VIIIa^***^, (*n* = 33)	Vermis VIIIa	0	−69	−41	21.04
	Lobule V	−4	−65	−9	7.14
		−15	−53	−20	9.27
	Lobule VI	28	−67	−19	10.17
Vermis VIIIb^*^, (*n* = 33)	Vermis VIIIb	0	−63	−42	30.17
	Crus I	−30	−84	−26	10.80
		−42	−55	−27	8.67
		51	−59	−26	8.52
	Lobules I–IV	−2	−48	−11	8.75
	Lobule VI	−21	−55	−21	8.73
Vermis IX^*^, (*n* = 33)	Vermis IX	0	−54	−34	30.28
	Lobule VIIIa	−25	−55	−49	9.52
	Vermis X	1	−48	−35	24.74
Vermis X^†^, (*n* = 33)	Brainstem	−10	−41	−45	8.12
	Lobules I–IV	13	−44	−18	7.71

Negative x-values indicate locations in the left hemisphere, while positive x-values indicate locations in the right hemisphere. All results are family-wise error corrected,

† p < 0.05,

* p < 0.01,

** p < 0.005,

*** p < 0.001.

**Table 3:** **MNI coordinates of the local maxima of brain regions showing functional connectivity with the lobules of the right cerebellar hemisphere**.

**Seed**	**Region**	**BA**	**MNI coordinates**			***T*-value**
			***x***	***y***	***z***	
Lobules I–IV^***^, (*n* = 33)	Anterior cingulate	32	2	26	30	11.95
	Angular gyrus	39	−52	−72	28	11.22
	Cingulate motor area	24	−4	−12	34	11.11
Lobule V^***^, (*n* = 33)	Post-central gyrus	43	56	−12	16	11.10
	Pre-central gyrus	4	−34	−18	−44	10.26
Lobule VI^***^, (*n* = 33)	Dorsal pre-motor cortex	6	−44	0	46	12.73
	Post-central gyrus	43	56	−14	16	12.41
	Dorsolateral prefrontal cortex	9	46	4	42	11.35
	Inferior frontal gyrus	47	50	18	−10	10.87
	Medial temporal gyrus	21	38	−4	−30	10.46
	Inferior parietal lobule	40	−50	−34	48	9.61
Crus I^***^, (*n* = 30)	Superior frontal gyrus (frontal eye fields)	8	−28	22	48	17.51
			30	18	48	11.05
	Medial frontal gyrus	10	0	54	−2	13.05
	Precuneus	7	2	−62	40	14.56
	Angular gyrus	39	−50	−62	34	13.33
	Inferior parietal lobule	40	46	−54	30	12.81
	Thalamus (dorsomedial nucleus)	–	−4	−22	6	12.05
	Brainstem	–	16	−28	−32	11.89
	Caudate	–	18	12	18	11.53
Crus II^***^, (*n* = 25)	Posterior cingulate	30	0	−48	20	13.56
	Superior frontal gyrus (frontal eye fields)	8	−12	34	50	13.41
	Medial frontal gyrus	10	−30	62	10	13.29
		11	−44	48	−14	10.90
	Thalamus (dorsomedial nucleus)	–	−6	16	10	11.30
	Inferior temporal gyrus	37	−60	−56	−8	12.89
	Inferior frontal gyrus	45	−52	22	22	12.06
	Precuneus	7	−4	−58	42	11.66
Lobule VIIb^†^, (*n* = 20)	Inferior temporal gyrus	37	−46	−56	−12	11.01
Lobule VIIIa^†^, (*n* = 19)	Posterior cingulate	23	8	−28	28	12.62
	Precuneus	7	−4	−70	46	13.26
Lobule VIIIb^†^, (*n* = 19)	–	–	–	–	–	–
Lobule IX^†^, (*n* = 23)	Posterior cingulate	31	4	−48	24	16.73
	Medial frontal gyrus	10	4	56	10	14.89
	Angular gyrus	39	46	−64	34	14.23
			−46	−68	39	13.44
	Thalamus (dorsomedial nucleus)	–	−4	18	8	13.94
	Superior frontal gyrus (frontal eye fields)	8	28	52	50	12.06
			−26	24	48	10.13
	Sub-callosal gyrus	25	−2	14	−12	11.78
Lobule X^†^, (*n* = 33)	Medial temporal gyrus	39	54	−64	22	9.97
	Posterior cingulate	29	−2	−38	20	9.69
	Precuneus	7	−2	−58	56	9.57
	Inferior parietal lobule	40	48	−64	46	9.02
	Thalamus (dorsomedial nucleus)	–	6	−20	16	8.74

Negative x-values indicate locations in the left hemisphere, while positive x-values indicate locations in the right hemisphere. All results are family-wise error corrected,

† p < 0.05,

*** p < 0.001.

N = 33, unless otherwise indicated.

**Table 4:** **MNI coordinates of the local maxima of brain regions showing functional connectivity with the lobules of the cerebellar vermis**.

**Seed**	**Region**	**BA**	**MNI coordinates**			***T*-value**
			***x***	***y***	***z***	
Vermis VI^**^, (*n* = 33)	Dorsolateral prefrontal cortex	46	34	36	26	12.65
		9	−32	46	30	9.59
	Inferior occipital gyrus	18	32	−94	−8	10.93
	Anterior cingulate cortex	32	−4	20	32	10.91
	Ventral pre-motor cortex	6	−52	2	6	10.37
	Posterior cingulate	23	2	−28	24	10.14
	Primary motor cortex	4	40	−2	44	10.08
	Inferior frontal gyrus	47	−24	22	−4	9.91
	Precuneus	7	−2	−50	60	9.88
Vermis Crus II^*^, (*n* = 33)	Anterior cingulate cortex	24	−14	−20	34	10.46
	Superior occipital gyrus	19	28	−70	32	10.23
	Brainstem	–	−8	−26	2	10.03
	Cuneus	19	−16	−86	24	9.42
	Precuneus	5/7	2	−44	62	8.86
Vermis VIIb^†^, (*n* = 33)	Thalamus (ventral anterior nucleus)	–	−8	−4	10	9.89
	Posterior cingulate cortex	23	6	−26	28	9.09
	Precuneus	7	−4	44	72	8.70
Vermis VIIIa^†^, (*n* = 33)	Paracentral lobule	6	−2	−32	76	9.17
	Precuneus	7	−2	−48	76	8.99
	Superior frontal gyrus	10	−30	46	26	8.68
Vermis VIIIb^†^, (*n* = 33)	Thalamus (dorsomedial nucleus)	–	2	−8	4	8.63
Vermis IX^†^, (*n* = 33)	Middle temporal gyrus	39	−48	−68	24	11.29
	Superior temporal gyrus	39	42	−52	28	11.04
	Superior frontal gyrus (frontal eye fields)	8	−28	20	48	10.55
	Middle frontal gyrus (frontal eye fields)	8	32	14	52	10.06
Vermis X^†^, (*n* = 33)	–	–	–	–	–	–

Negative x-values indicate locations in the left hemisphere, while positive x-values indicate locations in the right hemisphere. All results are family-wise error corrected,

† p < 0.05,

* p< 0.01,

** p < 0.005.

**Figure 1:**
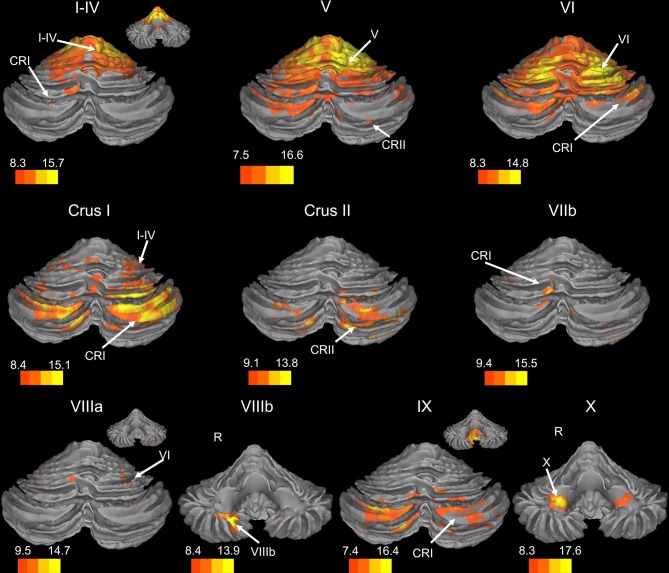
**Resting state connectivity of right hemisphere lobules within the cerebellum.** The color bars correspond to the colored shading and are indicative of the *t*-values at each region. The maps are thresholded such that only significant results are presented. In several cases, insets are provided to show the extent of the connectivity maps. In all cases, the right is presented on the right, and the left on the left side, except for the inferior views of the cerebellum. Here, the right hemisphere is presented on the left side and indicated by an “R.” CRI, Crus I; CRII, Crus II.

**Figure 2:**
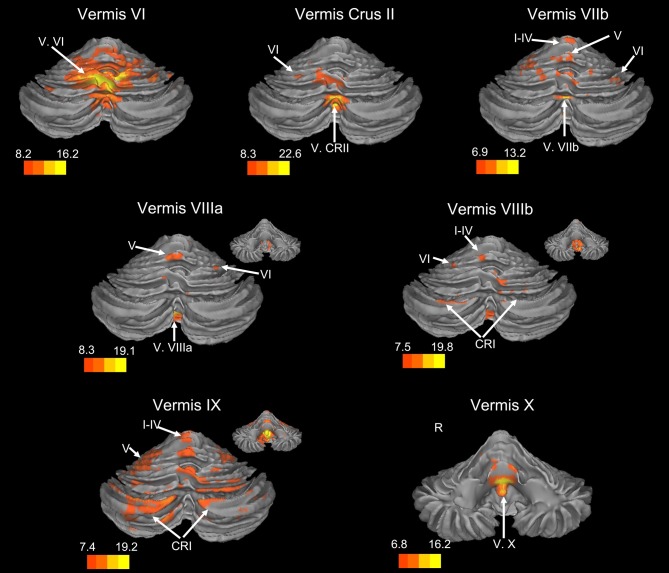
**Resting state connectivity of the cerebellar vermis within the cerebellum.** The color bars correspond to the colored shading and are indicative of the *t*-values at each region. The maps are thresholded such that only significant results are presented. In several cases, insets are provided to show the extent of the connectivity maps. In all cases, the right is presented on the right, and the left on the left side, except for the inferior views of the cerebellum. Here, the right hemisphere is presented on the left side and indicated by an “R.” CRI, Crus I; CRII, Crus II.

**Figure 3:**
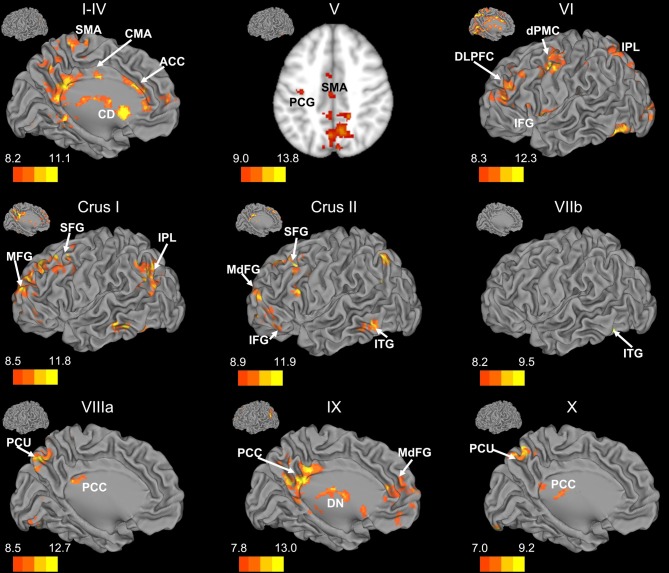
**Resting state cerebello-cortical connectivity maps of the right cerebellar hemisphere.** Whole-brain networks of the cerebellar lobules are displayed. The cross-laterality of the cerebellum resulted in networks largely in the left hemisphere from right cerebellar seeds. As such, we present only this hemisphere. Because of the location of the primary correlations with lobule V, a slice has been presented (*z* = 43). The color bars correspond to the colored shading and are indicative of the *t*-values at each region. The maps are thresholded such that only significant results are presented. Lobule VIIIb did not show any correlations with the whole brain and has therefore not been included here. CD, caudate; dPMC, dorsal pre-motor cortex; DLPFC, dorsolateral prefrontal cortex; DN, dorsomedial nucleus of the thalamus; IFG, inferior frontal gyrus; IPL, inferior parietal lobule; ITG, inferior temporal gyrus; MFG, middle frontal gyrus; MdFG, medial frontal gyrus; PCC, posterior cingulate cortex; PCU, precuneus; SFG, superior frontal gyrus; SMA, supplementary motor area.

**Figure 4:**
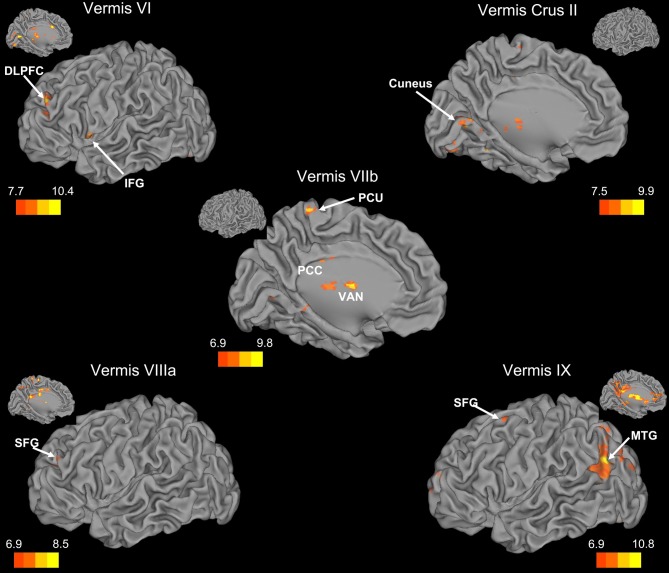
**Resting state cerebello-cortical connectivity maps of the cerebellar vermis.** Whole-brain networks of the cerebellar vermis are displayed on the left hemisphere. The color bars correspond to the colored shading and are indicative of the *t*-values at each region. Insets provide the additional view of the left hemisphere. The maps are thresholded such that only significant results are presented. Vermis X did not show any correlations with the whole brain and has therefore not been included here. DLPFC, dorsolateral prefrontal cortex; MTG, middle temporal gyrus; PCC, posterior cingulate cortex; PCU, precuneus; SFG, superior frontal gyrus; VAN, ventral anterior nucleus of the thalamus.

There was a general distinction between the lobules of the anterior cerebellum, made up of lobules I–IV and V, and the posterior cerebellum, made up of Crus I, Crus II, and lobules VIIb–X (Figures 1–4; results overlaid on cerebellar and brain surfaces using CARET; Van Essen et al., 2001). Within the anterior cerebellum, correlations were primarily with other anterior lobules, along with lobule VIIIb, largely indicative of the similar motor cortical targets of these regions. However, there were several weaker correlations between the anterior cerebellum with Crus I and Crus II. Not surprisingly, correlations between the anterior lobules and the cortex are primarily with motor and pre-motor cortical regions. Importantly, when we look at the correlations between lobule V and the primary motor cortex using a more relaxed threshold (family-wise error, *p* < 0.005), we see correlations with the majority of the motor strip. Lobule VI represented a transition region between the anterior motor networks and the posterior networks, more associated with cognitive and association regions. Within the cerebellum, this lobule was correlated strongly with *both* anterior and posterior cerebellar lobules, and in the whole brain, the correlations included both motor and cognitive/associative areas of cortex. In the posterior cerebellum, intracerebellar correlations were largely with other posterior lobules, though there were some small correlations with anterior lobules. However, lobules VIIIa and VIIIb were an exception in that they were primarily correlated with anterior cerebellar lobules. This is consistent with evidence for an additional motor representation in these lobules (Kelly and Strick, 2003; Stoodley and Schmahmann, 2009; Stoodley et al., 2012). Whole brain correlations of the posterior cerebellar lobules were largely with prefrontal and association cortices, and also indicated involvement in the default mode network. Somewhat surprisingly, we did not find correlations between Lobules VIIIa and VIIIb and motor cortical regions. Finally, the medial lobules of the cerebellar vermis were investigated. Connectivity patterns both within the cerebellum and the whole brain were diverse including motor, sensory, and association areas of the cortex. Our confirmatory analysis looking at a small seed in the left primary motor cortex demonstrated our ability to successfully reproduce these networks. There were correlations between the motor cortex and right lobule V, lobules I–IV, and the right dorsal dentate nucleus (consistent with what would be expected based on Dum and Strick, 2003).

**Figure 5:**
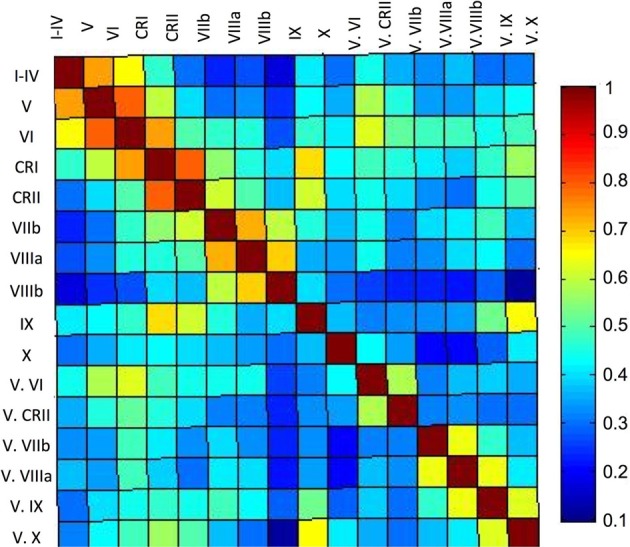
**Correlation matrix of the lobules of the right cerebellar hemisphere and vermis.** The correlation matrix based on the average timecourses of the lobules of the right hemisphere and vermis indicates primarily local correlations, with some cases of correlations across greater distances, perhaps based on functional similarities. The color bar indicates the *r*-values presented in the matrix.

Finally, our correlation matrix based on the average timecourse in each lobule revealed higher correlations (Figure 5) between spatially adjacent regions, with several exceptions. While lobules I–IV, V, and VI are all strongly correlated, lobule VI is also correlated more strongly with Crus I, consistent with the notion that it is a transition zone between motor regions in the anterior cerebellum and non-motor regions in the posterior cerebellum. Also interesting is the correlations between Crus I and Crus II and lobule IX. These regions have been purported to be associated with the default mode network (Buckner et al., 2011) and interestingly, are correlated in the cerebellum over a reasonable distance (they are not directly adjacent lobules). The last notable pattern is that of strong correlations between a hemispheric lobule and its vermal counterpart. However, these are anatomically adjacent regions, consistent with the pattern of stronger correlations between spatially adjacent regions.

### SOM results

The SOM analysis divided the cerebellar resting-state data into 20 clusters. Table 5 provides a breakdown of the lobular coverage in each cluster. While there were several clusters that were restricted in their extent to one lobule (clusters 3, 8, 10, 14, and 18, found in lobule VI and Crus I), the other 15 clusters included regions extending across multiple lobules. It is also worth noting that in those clusters that were restricted only to one lobule, they covered a small subdivision of that particular lobule.

**Table 5:** **Lobular mappings of the SOM algorithm clusters within the cerebellum**.

**SOM cluster**	**Lobular coverage**	**Bilateral? Y/N**
1	L. and R. VIIb	Y
	L. Crus I	
2	R. VI	N
	Crus I	
3	R. Crus I	N
4	L. and R. I–IV	Y
	L and R. V	
	L. VI	
5	R. V	N
	R. VI	
6	R. Crus I	Y
	L. Crus I	
	R. Crus II	
7	L. and R. Crus I	Y
	L. and R. Crus II	
8	L. Crus I	N
9	L. VIIIa	N
	L. Crus II	
	L. and R. Crus I	
10	R. VI	N
11	L. and R. Crus II	Y
	L. and R. VIIIa	
	L. and R. VIIIb	
	L. and R. IX	
12	L. and R. Crus I	Y
	L. and R. Crus II	
	L. and R. IX	
	Vermis VIIIa	
	Vermis VIIIb	
	Vermis IX	
13	L. and R. Crus I	Y
	L. VI	
14	R. Crus I	N
15	L. and R. VI	Y
	L. Crus I	
	L. and R. X	
16	L. and R. V	Y
	L. and R. VI	
17	L. and R. Crus II	Y
	R. VIIb	
	L. VIIIa	
18	R. Crus I	N
19	L. and R. Crus II	Y
20	L. and R. I–IV	Y
	L. and R. V	
	L. VI	
	L. and R. VIIIa	
	L. and R. IX	
	Vermis VI	
	Vermis Crus II	
	Vermis VIIIa	
	Vermis VIIIb	

The extent of the clusters was determined using the SUIT atlas (Diedrichsen, 2006; Diedrichsen et al., 2009).

**Figure 6:**
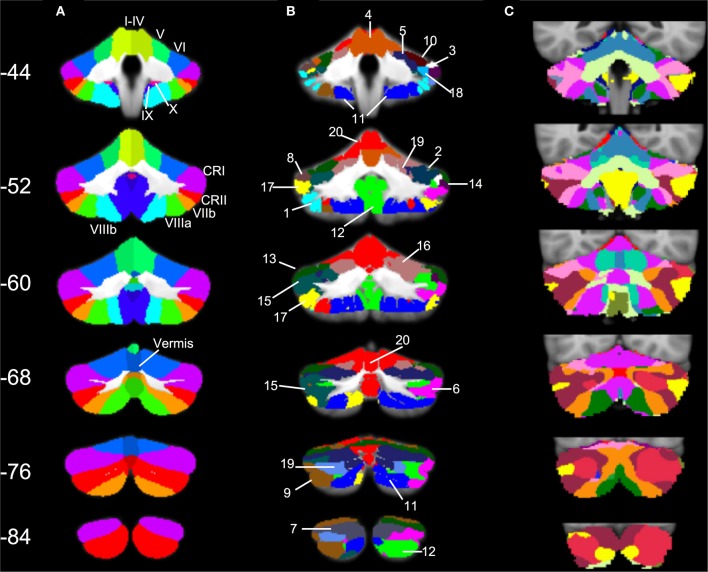
**SOM results in comparison to cerebellar anatomy and cerebellar parcellation based on cortical networks. (A)** Anatomical masks from the SUIT atlas, with labeled lobules (Diedrichsen, 2006; Diedrichsen et al., 2009) used in our lobular analyses overlaid on the SUIT cerebellum. **(B)** The twenty clusters from our SOM algorithm overlaid onto the SUIT cerebellum. The numbers correspond to the clusters as described in Table 5. **(C)** The 17-network cortical solution in the cerebellum (from Buckner et al., 2011). Coronal slices from anterior (*Y* = −44) to posterior (*Y* = −84).

Three particularly striking patterns can be seen in our SOM results. First, we found one large cluster (cluster 20) that included at least a portion of all of the cerebellar lobules known to play a role in motor functions. This bilateral cluster was predominant in the anterior cerebellum including lobules I–VI, but also included lobule VIIIa and all of the anterior vermis. Second, there was a large posterior cluster that included the most inferior portions of lobules VIIIa and b, lobule IX, and Crus II, all bilaterally. Finally, it is notable that Crus I was subdivided across multiple clusters. Several of these clusters (3 and 8, and 14 and 18) appear to be mirrored in the two homologues, though they clustered separately. Figure 6 provides a side-by-side comparison of the anatomical masks used in our lobular connectivity analysis (Diedrichsen, 2006; Diedrichsen et al., 2009), our SOM clusters, and the seventeen-network parcellation based on the cortex by Buckner and colleagues (2011). Similarities and differences between these methods will be addressed further in the discussion.

**Figure 7:**
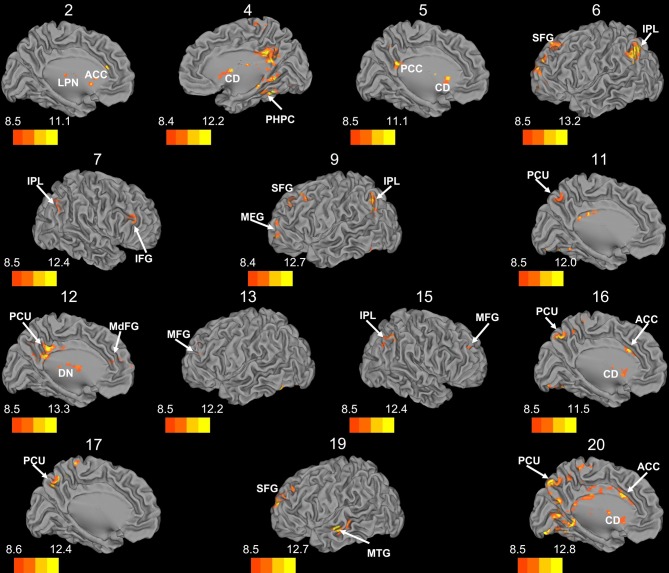
**Whole brain connectivity of the self-organizing map clusters.** Results are presented on the left hemisphere, with the exception of clusters 4, 7, and 15, which are presented on the right. Clusters 2, 4, 5, 6, 11, 12, 16, 17, and 20 are medial views. Clusters 3, 8, 14, and 18 did not show suprathreshold clusters, while clusters 1 and 10 were only correlated with regions of the cerebellum, so these maps are not included. The color bars correspond to the colored shading and are indicative of the *t*-values at each region. The maps are thresholded such that only significant results are presented. MFG, middle frontal gyrus; MdFG, medial frontal gyrus; IPL, inferior parietal lobule; SFG, superior frontal gyrus; ACC, anterior cingulate; PCC, posterior cingulate; PCU, precuneus; SMA, supplementary motor area; MPFC, medial prefrontal cortex; CD, caudate; LPN, lateral posterior nucleus of the thalamus; MTG, middle temporal gyrus.

Whole brain connectivity using the 20 SOM clusters as masks revealed specific networks between sub-regions in the posterior cerebellum, while the large anterior cluster (20) showed a non-specific network, with some pre-motor regions, though no correlations with the primary motor cortex. Please see Table 6 for regions of peak correlation within the cortex for all of the clusters. Consistent with prior work, the sub-regions within Crus I and Crus II produced distinct networks with regions of the prefrontal cortex. The specificity of these networks is demonstrated in Figure 7. Somewhat surprisingly, the large anterior cluster showed no connectivity with regions of the primary motor cortex, as expected and demonstrated when the component lobules were investigated individually. The small, mirrored clusters within Crus I (clusters 3 and 8, and 14 and 18) also revealed no connectivity across the whole brain, or within the cerebellum itself (which is unsurprising given their independence demonstrated in the SOM algorithm). The large posterior cluster encompassing Crus I and II, posterior vermis, and lobule IX was correlated with structures that make up the default mode network, consistent with the findings of Buckner et al. (2011). Finally, across multiple clusters there were strong correlations with the basal ganglia (both caudate and putamen), highlighting potential interactions between these two structures, and may be reflective of anatomical connections between the two, supported by recent evidence of anatomical connections between these structures (Hoshi et al., 2005; Bostan et al., 2010).

**Table 6:** **MNI coordinates of the local maxima of brain regions showing functional connectivity with the clusters resulting from our SOM algorithm**.

**Seed**	**Region**	**BA**	**MNI coordinates**			***T*-value**
			***x***	***y***	***z***	
Cluster 2	Thalamus (lateral posterior nucleus)	–	−14	−22	12	12.77
	Anterior cingulate cortex	32	−2	34	24	11.84
Cluster 4	Parahippocampal gyrus	34	16	−10	−20	18.92
	Middle frontal gyrus	6	2	−26	78	14.82
	Caudate	–	−4	12	−4	13.92
Cluster 5	Posterior cingulate cortex	29	6	−44	16	12.44
	Caudate	–	10	2	6	12.15
		–	−6	18	2	10.73
Cluster 6	Angular gyrus	39	44	−60	34	15.78
	Lateral parietal sulcus/inferior parietal lobule	7	−42	−66	48	15.25
	Precuneus	31	14	−44	34	14.36
	Superior frontal gyrus	10	−6	70	0	13.89
	Superior frontal gyrus (frontal eye fields)	8	−6	38	52	12.97
		8	26	32	52	12.13
	Caudate	–	−8	29	−2	12.82
	Medial frontal gyrus	11	6	40	−12	12.53
Cluster 7	Putamen	–	12	2	6	12.97
	Caudate	–	−10	12	10	11.70
	Inferior frontal gyrus	9	54	22	26	11.59
	Inferior parietal lobule	40	48	−48	28	10.98
Cluster 9	Lateral parietal sulcus	7	42	−62	50	17.29
	Middle frontal gyrus	10	−30	56	−6	14.26
			28	60	8	12.60
		8	−36	22	40	12.44
	Superior frontal gyrus (frontal eye fields)	8	26	32	52	12.04
	Medial temporal gyrus	21	64	−38	−10	11.80
Cluster 11	Precuneus	7	0	−66	44	10.56
Cluster 12	Inferior parietal lobule	7	−40	74	−48	16.13
	Precuneus	7	10	−48	32	15.76
	Supramarginal gyrus	39	56	−58	30	13.55
	Medial frontal gyrus	10	−2	56	10	12.51
	Thalamus (dorsal medial nucleus)	–	−4	−16	8	12.50
Cluster 13	Precuneus	7	−2	−54	54	20.08
	Middle frontal gyrus	10	34	56	18	12.83
	Superior frontal gyrus	10	−24	46	22	12.24
	Angular gyrus	39	−42	−58	32	12.00
	Anterior cingulate cortex	32	2	26	30	11.80
Cluster 15	Posterior cingulate	23	−2	28	26	16.48
	Thalamus	–	6	−24	4	15.77
	Anterior cingulate cortex	32	6	32	28	15.55
	Superior frontal gyrus	10	−26	60	−2	15.11
	Inferior parietal lobule	7	38	−60	44	12.84
	Middle frontal gyrus	10	34	36	24	12.75
		8	−30	22	40	11.97
Cluster 16	Anterior cingulate cortex	32	22	0	26	14.88
			−4	20	30	12.60
	Caudate	–	−16	−12	22	12.89
	Putamen	–	28	−8	8	12.84
	Thalamus	–	8	0	6	12.22
	Precuneus	7	−2	−48	58	11.76
Cluster 17	Precuneus	7	−2	−58	58	15.12
Cluster 19	Angular gyrus	39	46	−56	34	16.04
	Precuneus	31	12	−48	34	15.99
	Superior frontal gyrus	10	−4	66	24	15.68
	Medial temporal gyrus	21	−60	−14	−8	14.24
Cluster 20	Nucleus accumbens	–	28	−6	−12	16.09
	Dorsal pre-motor cortex	9	48	4	40	13.30
	Superior temporal gyrus	22	−54	2	4	13.28
		38	52	14	−10	12.47
	Medial frontal gyrus	32	−4	18	36	13.18
	Caudate	–	20	18	16	12.92
	Middle frontal gyrus	9/46	36	42	22	12.44
	Putamen	–	−30	−14	−4	11.31

Negative x-values indicate locations in the left hemisphere, while positive x-values indicate locations in the right hemisphere. All results are family-wise error corrected, p < 0.05. Clusters 1 and 10 only displayed within cluster correlations in the cerebellum. Clusters 3, 8, 14, and 18 had no suprathreshold clusters.

### Consistency and reproducibility

Applying the SOM algorithm on the data results in 100 clusters, as specified in the algorithm. Performing the superclustering 1000 times on the full data set resulted in 21 (±2.8) clusters (mean ± std). Thus, our presented result of 20 clusters is taken to be a representative sample, with very good stability. Additionally, in performing the SOM analysis on separate subgroups of *n* = 10 and *n* = 9 subjects, with 100 iterations of superlclusering, resulted in 22 (±2.4) and 22 (±3.2) clusters, respectively. Thus, even at smaller sample size, the number of clusters we find is consistent.

## Discussion

Here, we provide a detailed mapping of the resting state networks of the human cerebellum created using both lobular anatomically-driven and self-organizing map-driven approaches. Our analyses revealed connectivity networks within the cerebellum, largely segregated between anterior and posterior lobules, likely due to overlapping cortical targets for these regions. Analyses in the whole brain revealed distinct networks associated with each lobule. The use of a SOM algorithm allowed us to investigate the networks of the cerebellum based on their functional similarities. We found that the resting-state data of the cerebellum divided into 20 clusters, which combine and extend across some lobules, while subdividing others into smaller functional units. The whole-brain correlation analyses demonstrated distinct networks between the posterior cerebellum and anterior prefrontal and parietal regions of the cerebral cortex, while the large clustering of the anterior lobules resulted in a smoothing effect, blurring the specific lobular connectivity seen in individual anterior lobules. Comparisons between the two methods will be addressed below.

### Lobular networks of the human cerebellum

To our knowledge, this is the first resting state study of the cerebellum to investigate resting-state networks within the cerebellum itself. Using the individual lobules as masks, our analyses indicated a rough distinction between the networks of the anterior cerebellum (lobules I–VI) and the posterior cerebellum (Crus I–Lobule X). The majority of the correlations for individual lobules occurred in mostly the same cerebellar region, though Lobule VI appeared to be a transition area between the two regions, encompassing a wide variety of lobules in its network. Similarly, lobules VIIIa and VIIIb, which are known to have an additional motor representation (Kelly and Strick, 2003; Stoodley and Schmahmann, 2009; Stoodley et al., 2012) showed strong correlations with the anterior cerebellum. However, in most cases, there were some small correlations that spanned across this anterior/posterior division. For example, Crus I was primarily correlated with other posterior lobules, though there was also a region of correlation with Lobules I–IV.

Further analysis using a correlation matrix based on the average timecourse in each lobule further supports these regional subdivisions, as the more anterior regions of the cerebellum were more strongly correlated with one another, as were the lobules of the vermis. Also of note were the correlations between the vermal lobules and their hemispheric neighbors. Typically, these were high, indicating a general functional similarity, despite the anatomical boundary. In general, there seem to be correlations within the cerebellum that are in many ways consistent with the rough functional demarcations of the anterior and the posterior cerebellum.

In considering these resting-state networks within the cerebellum, it is important to consider the underlying neurophysiology of the structure. The cerebellum is thought to communicate with the cerebral cortex through closed-loop circuits (Kelly and Strick, 2003; for a review see Ramnani, 2006). The cerebellar hemispheres synapse onto the deep cerebellar nuclei, and from there, projections reach the thalamus, and finally the cerebral cortex. Given the segregated loops of the cerebellum, the intracerebellar resting state networks are likely due to similar cortical targets of these cerebellar regions, or additional processing that occurs in the cortex or thalamus. Regions of the cerebellum that are correlated in the resting state thus may be due to similar cortical targets of closed-loop circuits. Nonetheless, these resting state cerebellar networks provide further insight into the functional organization of the structure.

When our findings were extended to the whole brain, we found distinct networks for individual lobules. However, the anterior and posterior distinction of the cerebellum translated into whole-brain networks encompassing motor cortical regions, and cognitive and association regions, respectively. The motor and cognitive distinction seen both within the cerebellum, and also in the whole brain, is consistent with tract-tracing data in non-human primates indicating distinct loops between motor and prefrontal regions of cortex with the anterior and posterior cerebellum, respectively (Kelly and Strick, 2003). Furthermore, this is in accordance with human neuroimaging data indicating a functional topography within the cerebellum (Stoodley and Schmahmann, 2009; Stoodley et al., 2012) and patient studies dissociating between the motor and cognitive functions of the anterior and posterior cerebellum (Schmahmann and Sherman, 1998; Schmahmann et al., 2009).

Within the human cerebellum, the vermis has been relatively understudied using resting state fcMRI. Somewhat surprisingly, we found that the networks of the vermis extended beyond just motor regions. The resting state networks of the vermal lobules also included regions in the prefrontal cortex, as well as those associated with auditory and visual processing. While animal data have demonstrated direct connections between the motor cortex and the vermis (Coffman et al., 2011) and our correlations between the vermis and these regions are expected, the more widespread cortical correlations are somewhat surprising. However, when we consider the role of the cerebellar vermis (spinocerebellum) in maintaining posture (Ouchi et al., 1999, 2001; Sullivan et al., 2010), the correlations with other sensory regions (visual and auditory cortices) are plausible. Sensory integration across domains may be key for the maintenance of posture and balance, and as such, these cortical regions are a part of these networks of the cerebellar vermal lobules.

Overall, our lobular mappings indicate that the cerebellum is involved in a variety of resting state networks segregated by function (motor and cognitive). Notably, Buckner and colleagues (2011) demonstrated mirrored representations of the cortex, and found that most of the cerebellum is correlated with association areas of the cortex. Our results are indeed consistent with this latter notion. However, though our results are broadly consistent with the notion of a mirrored representation of the cortex, we report no correlations between Lobules VIIIa and VIIIb and motor cortical regions. It is unclear why our analyses did not reveal these networks given that these correlations have been demonstrated previously (Krienen and Buckner, 2009), though within the cerebellum lobules VIIIa and VIIIb are correlated with anterior lobules, supporting a role for these lobules in motor processing, as opposed to cognitive. It may be that our smaller sample size in these regions decreased our power to detect such correlations, or there is a decrease in signal-to-noise ratio in this region. Finally, we do report that Lobule IX is correlated with anterior prefrontal regions, in a pattern similar to that of Crus I, which supports the possibility of a third cortical representation, which was proposed by Buckner et al. (2011).

### Self-organizing-map algorithm

Our parcellation of the cerebellum using a SOM algorithm resulted in 20 clusters. Across both hemispheres, the cerebellum is made up of 34 distinct anatomical lobules (28 total masks within the SUIT atlas). Though the SOM algorithm grouped the cerebellar timecourses to 20 clusters, it did not do so following lobular boundaries. Several lobules, particularly those related to motor function in the anterior cerebellum and lobule VIIIa, clustered bilaterally into one large grouping. There was a similar large cluster in the posterior inferior cerebellum as well. Conversely, Crus I was subdivided into multiple smaller regions, several of which were unilateral, though closely mirrored in the two hemispheres. This was also the case for lobule VI and Crus II, though not to the same degree.

Visual comparison of the results of our SOM analyses with the 17-network solution presented by Buckner and colleagues (2011) reveals some areas of similarity, but also some key differences. Most notably is the number of regions. Our SOM approach starting in the cerebellum resulted in 20 regions within the cerebellum. The extension of our large cluster encompassing anterior cerebellum and lobule VIII is similar to a combination of several of the anterior networks presented by Buckner and colleagues (2011), though our cluster extends further in the anterior direction (for comparison see our red regions and Buckner's in violet, particularly on *Y* = −60 and −68). Seen most prominently in slice *Y* = −52 is the fact that while Buckner and colleagues (2011) have multiple regions in the inferior cerebellum, we have one large cluster extending bilaterally, and continuously (blue). Conversely, in Crus I we demonstrate a greater subdivision with respect to that presented by Buckner and colleagues (2011). Taken as a whole, while there is some consistency across these approaches, our results reveal additional cerebello-cortical networks that were not previously defined using a cortically based approach. As we speculated previously, functional regions of the cerebellum may be correlated with specific, small cortical regions. The further subdivision of the posterior cerebellum in particular support this claim, and highlight the utility of taking a cerebellar-driven approach to understanding the functional networks of the structure.

The presence of a large cluster collapsing across lobules with known motor function highlights the similarity of the processing that occurs in these regions. Individually, these regions are correlated with motor and pre-motor cortical areas. Thus, the overall similarity in the resting-state signal was likely such that they clustered together in our SOM. However, at the whole-brain level when connectivity was assessed using this region as a mask, there were diffuse correlations that did not encompass the primary motor cortex. These regions included the caudate, putamen, middle frontal gyrus, medial frontal gyrus, dorsal pre-motor cortex, nucleus accumbens, and the superior temporal gyrus. Likely, the averaging across the entire cluster resulted in a smoothing of the signal to such an extent that differentiating between motor and pre-motor networks was not possible. Our lobular analyses showed specific correlations with motor and pre-motor regions, and Buckner and colleagues (2011) were able to further subdivide this region somatotopically using resting state connectivity, indicating that there are specific networks in the anterior cerebellum. Notably, our lobular analyses and results from other resting-state studies of the cerebellum (Krienen and Buckner, 2009) indicate that the resting state motor networks are lateralized. The bilateral nature of this cluster, and the averaging across the two hemispheres may have also contributed to the lack of specific motor and pre-motor correlations. Thus, while within the cerebellum our SOM analyses indicate similarities in these regions resulting in the large grouping, the specific lobules are more informative for investigating resting state networks of the anterior cerebellum. This underscores the utility of taking a lobular approach to investigating cerebello-cortical networks associated with motor function.

The subdivision of Crus I into multiple smaller functional clusters may reflect the heterogeneity of cognitive processing. These subdivisions were correlated with distinct regions of prefrontal and temporal cortex, along with parietal cortex. These findings are in line with the distinct networks shown by Buckner and colleagues (2011) when seeding in specific regions of posterior cerebellum. Also of note are the mirrored clusters in this lobule. While, the clusters covered essentially same spatial regions in both hemispheres, they were divided by our SOM algorithm into distinct clusters. However, these clusters exhibit no correlations with the cortex when used as masks for resting-state analyses. It may be the case that these clusters are important for intra-cerebellar processing, and may represent local processing of information related to cognition. In sum, our results indicate the utility of subdividing the posterior cerebellum using this data driven-approach as it reveals distinct sub-regions that are a part of unique whole-brain networks. This functionally-defined parcellation may be particularly relevant in aging or patient populations, where anatomical and functional boundaries may be altered.

Taken in the greater context of resting state connectivity studies of the human cerebellum, our study contributes several important findings. As we noted previously, this is the first study to our knowledge to also investigate the resting state networks within the cerebellum. Additionally, with the exception of the recent work by Buckner and colleagues (2011), resting state studies of the human cerebellum have focused on small regions within the cerebellum, or they have looked at correlations between small regions or networks in the cerebral cortex and the cerebellum. Here, we have provided a detailed mapping of resting-state cerebello-cortical networks using both lobular anatomical regions of interest, and those defined by a SOM algorithm. Though, we have likely just scratched the surface given that we averaged across multiple participants, and individual differences can provide great insight into our understanding of brain function (Kanai and Rees, 2011), this averaging was important in this first step to better understand the functional organization of the cerebellum with respect to anatomy.

Our anatomical mapping provides an atlas of resting state-networks based on cerebellar anatomy. Such an atlas may be important for future investigations in patient populations that have damage to specific lobules or regions of the cerebellum that might result in changes in functional connectivity. We have also provided a functional parcellation of the cerebellum, and subsequent resting state mapping. Most importantly, we found that in general, functional organization of the human cerebellum does not map onto lobular anatomy. While, the lobular organization of the cerebellum creates distinct anatomical regions, functionally, there is little overlap. Indeed, similar claims that lobular boundaries do not indicate functional boundaries have been put forth based on molecular and anatomical studies (Apps and Hawkes, 2009).

## Conclusions

Our approach of investigating the resting state networks of the human cerebellum using an anatomical lobular approach revealed distinct networks of the individual lobules, though a broad distinction between the anterior and posterior cerebellum was noted. Lobules of the anterior cerebellum were more strongly correlated with motor cortical regions, whereas those of the posterior cerebellum were correlated with prefrontal and parietal cortices. This approach also demonstrated correlations between the vermis and both motor-and non-motor cortical areas. Using a SOM algorithm to sub-divide the cerebellum functionally resulted in a clustering across anterior lobules and the posterior inferior cerebellum. However, there was also greater subdivision in lobules Crus I and Crus II. While this approach was useful for differentiating additional networks in the large lobules of the posterior cerebellum, it blurred the distinct networks of the anterior lobules. Thus, a lobular approach may be more appropriate for investigating motor networks of the cerebellum, while a self-organizing functional approach provides more detailed information about the networks of the large posterior lobules. The differences between the cerebellar SOM approach and a cortically based division of the cerebellum indicate that there are further subdivisions within the cerebellum that should be considered when investigating this structure. Finally, our results indicate that the anatomical boundaries between the lobules do not indicate functional boundaries, as some regions are functionally similar across lobules, while other lobules are further divided into additional functional sub-regions.

### Conflict of interest statement

The authors declare that the research was conducted in the absence of any commercial or financial relationships that could be construed as a potential conflict of interest.
